# Supplementary material for: Empirical methods for controlling false positives and estimating confidence in ChIP-Seq peaks
Source: BMC Bioinformatics. 2008 Dec 5;9:523. doi: 10.1186/1471-2105-9-523 (PMC2628906; doi:10.1186/1471-2105-9-523)
Supplement: Additional File 1 — A variety of html documents from the USeq web site detailing the available applications, their best usage, output file type descriptions, command line menus, etc. [file 1471-2105-9-523-S1.zip › USeqUserGuides/JavaDoc.zip!/JavaDoc/edu/utah/seq/data/class-use/Point.html]

Uses of Class edu.utah.seq.data.Point


|  |  |  |
| --- | --- | --- |
| **Overview** \| **Package** \| **Class** \| **Use** \| **Tree** \| **Deprecated** \| **Index** \| **Help** | |  |
| PREV   NEXT | **FRAMES**    **NO FRAMES**     **All Classes** |  |


---


## **Uses of Class edu.utah.seq.data.Point**

| Packages that use Point | |
| --- | --- |
| **edu.utah.seq.data** | Helper classes for working with binary PointData, EnrichedRegions, etc. |
| **edu.utah.seq.parsers** | Parsers for converting sequencing data into binary PointData, xxx.bar.zip files. |

Uses of Point in edu.utah.seq.data

| Subclasses of Point in edu.utah.seq.data | |
| --- | --- |
| `class` | `InfoPoint` |

| Methods in edu.utah.seq.data that return Point | |
| --- | --- |
| `Point[]` | `EnrichedRegion.getControlPoints()` |
| `Point[]` | `EnrichedRegion.getTreatmentPoints()` |
| `static Point[]` | `Point.makePoints(int[] positions, float[] scores)` |
| `Point[]` | `PointData.windowSum(int startBp, int stopBp, int halfWindowSize)` |

| Methods in edu.utah.seq.data with parameters of type Point | |
| --- | --- |
| `static PointData` | `Point.extractPositionScores(Point[] p)` |
| `void` | `EnrichedRegion.setControlPoints(Point[] controlPoints)` |
| `void` | `EnrichedRegion.setTreatmentPoints(Point[] treatmentPoints)` |

Uses of Point in edu.utah.seq.parsers

| Methods in edu.utah.seq.parsers that return Point | |
| --- | --- |
| `Point` | `ElandExportLine.fetchPoint()` |

---


|  |  |  |
| --- | --- | --- |
| **Overview** \| **Package** \| **Class** \| **Use** \| **Tree** \| **Deprecated** \| **Index** \| **Help** | |  |
| PREV   NEXT | **FRAMES**    **NO FRAMES**     **All Classes** |  |


---
